# A monoclinic semiorganic molecular crystal GUHP for terahertz photonics and optoelectronics

**DOI:** 10.1038/s41598-021-02862-3

**Published:** 2021-12-06

**Authors:** Anton Sinko, Peter Solyankin, Aleksey Kargovsky, Vera Manomenova, Elena Rudneva, Natalia Kozlova, Natalia Sorokina, Fedor Minakov, Sergei Kuznetsov, Nazar Nikolaev, Nikolay Surovtsev, Ilya Ozheredov, Alexey Voloshin, Alexander Shkurinov

**Affiliations:** 1 Faculty of Physics, Lomonosov Moscow State University, GSP-1, 1-2 Leninskiye Gory, Moscow, Russia 119991; 2 Institute on Laser and Information Technologies - Branch of the Federal Scientific Research Center “Crystallography and Photonics” of the Russian Academy of Sciences, Svyatoozerskaya Str. 1, Shatura, Russia 140700; 3 Penza State University, Krasnaya Str. 40, Penza, Russia 440026; 4 Shubnikov Institute of Crystallography of the Federal Scientific Research Center “Crystallography and Photonics” of the Russian Academy of Sciences, Lenin Ave. 59, Moscow, Russia 119333; 5 Institute of Automation and Electrometry of the Siberian Branch of the Russian Academy of Sciences, Academician Koptyug Ave. 1, Novosibirsk, Russia 630090; 6 Novosibirsk State University, Pirogova Str. 2, Novosibirsk, Russia 630090; 7 Rzhanov Institute of Semiconductor Physics of the Siberian Branch of the Russian Academy of Sciences, Novosibirsk Branch “TDIAM”, Academician Lavrentyev Ave. 2/1, Novosibirsk, Russia 630090

**Keywords:** Optical physics, Optical materials and structures, Optical physics

## Abstract

In this paper we describe the properties of the crystal of guanylurea hydrogen phosphite (NH$_2$)$_2$CNHCO(NH$_2$)H$_2$PO$_3$ (GUHP) and propose its application in terahertz photonics and optoelectronics. GUHP crystal has a wide window of transparency and a high optical threshold in the visible and NIR spectral regions and narrow absorption bands in the terahertz frequency range. The spectral characteristics of absorption and refraction in the THz range were found to be strongly dependent on crystal temperature and orientation. Computer simulations made it possible to link the nature of the resonant response of the medium at THz frequencies with the molecular structure of the crystal, in particular, with intermolecular hydrogen bonds and the layered structure of the lattice. The possibility of application of the crystal under study for the conversion of femtosecond laser radiation from visible an NIR to terahertz range was demonstrated. It was shown that dispersion properties of the crystal allow the generation of narrow band terahertz radiation, whose spectral properties are determined by conditions close to phase matching. The properties of the generated terahertz radiation under various temperatures suggest the possibility of phonon mechanism of enhancement for nonlinear susceptibility of the second order.

## Introduction

The research of new organic nonlinear optical crystals is a prime direction towards the development of efficient ultra-fast optical and optoelectronic devices due to their terahertz (THz) spectral range applications. In the past decades, nonlinear dielectric crystals, like ZnTe^1^, GaP^2^, LiNbO$_3$^3^, and many others^4^, were investigated as THz sources and receivers. Moreover, new efficient nonlinear organic crystals were created in view of THz studies, like DAST^5^, OH1^6^ (see review of these crystals in^7^). Presently, all these crystals are widely used in THz science and technology, despite the fact that each of them is not devoid of drawbacks. For example, ZnTe and GaP are suitable for sensing the spectral region below 2–3 THz, but they exhibit rather weak nonlinear optical coefficients. Extremely powerful THz signals are generated with LiNbO$_3$ sources, but only in Cherenkov-like or quasi-phase-matching-like schemes. Finally, organic crystals are the most promising ones due to the lower complexity of the crystal growth technology and their high nonlinear optical coefficients^8^. However, up to now, most of these organic crystals, like DAST, have shown high absorption at THz frequencies, which reduces their effectiveness and the components required for growth are usually rather expensive.

In most of the published studies, THz absorption resonances in crystals are considered as a drawback because they weaken THz generation efficiency. However, in the frequency range of the resonance, the spectrum of the refractive index shows a shape that resembles the frequency derivative of the absorption peak, as determined through the Kramers–Kronig transformation. As THz generation efficiency is governed by the difference between THz refractive index and the group index at the laser frequency, such difference could be minimized (approached phase-matching) or even made null (perfect phase-matching) at the frequency of resonance. Therefore, one expects a strong increase of THz generation efficiency close to the resonance frequency. This is possible if the influence of phase-matching process exceeds the effect of loss near the resonance. This occurs when the resonance exhibits a relatively narrow absorption band. It is also important to take into account a possible enhancement of second order susceptibility near the resonance area^9^. This factor becomes relevant in low-symmetry crystal structures. In this case, these phenomena will permit the generation of narrow-band THz radiation without a complex experimental setup. Moreover, dealing with phononic resonance, the frequency of the generated THz radiation can be tuned by adjusting the crystal temperature, since the spectral properties of the generated radiation are determined by the linear optical properties of the crystal, which depend on the temperature^10–12^.

Recently, papers related to the demonstration of such a narrow-band THz generation have begun to appear. Crystals of low symmetry (triclinic and monoclinic classes) often exhibit most of aforementioned complex and interesting properties. For example, one of the absorption peaks of a monoclinic Seraphinite crystal^13^ has a Q-factor about 8, which is relatively high, as will be shown below. The well-known DAST crystal also shows a lot of resonance absorption features in the THz spectrum, but all of them have rather wide peaks, so they are very unlocalized. Recently the nonorganic BaGa$_4$Se$_7$ crystal showed^14–16^ an incredible ability to generate narrowband THz radiation, but it did not demonstrate any variability in spectral tuning in the low THz spectral range (0.2–2 THz) which is the most interesting due to its intermediate position on the border of modern fast electronics. There are other papers^17,18^ describing the generation of terahertz radiation induced by the excitation of coherent phonons, which shows that this topic is relevant and interesting. In accordance with the above, we believe that the combination of the advantages of organic and inorganic crystalline media will be most promising when searching for media with narrow resonances in the absorption as well as in the generation. Thus, the semi-organic crystal, such as the recently grown guanylurea hydrogen phosphate (NH$_2$)$_2$CNHCO(NH$_2$)H$_2$PO$_3$ (GUHP) crystal^19^, may be the optimal choice. GUHP crystal has a wide window of transparency in the visible and NIR spectral regions and narrow absorption bands in the terahertz frequency range, which can be attributed to phonon response at THz frequencies^20^. Moreover, this crystal has already shown itself to be an effective nonlinear optical source for second harmonic generation^21^.

## Results

### Terahertz and Raman spectroscopy results

**Figure 1:**
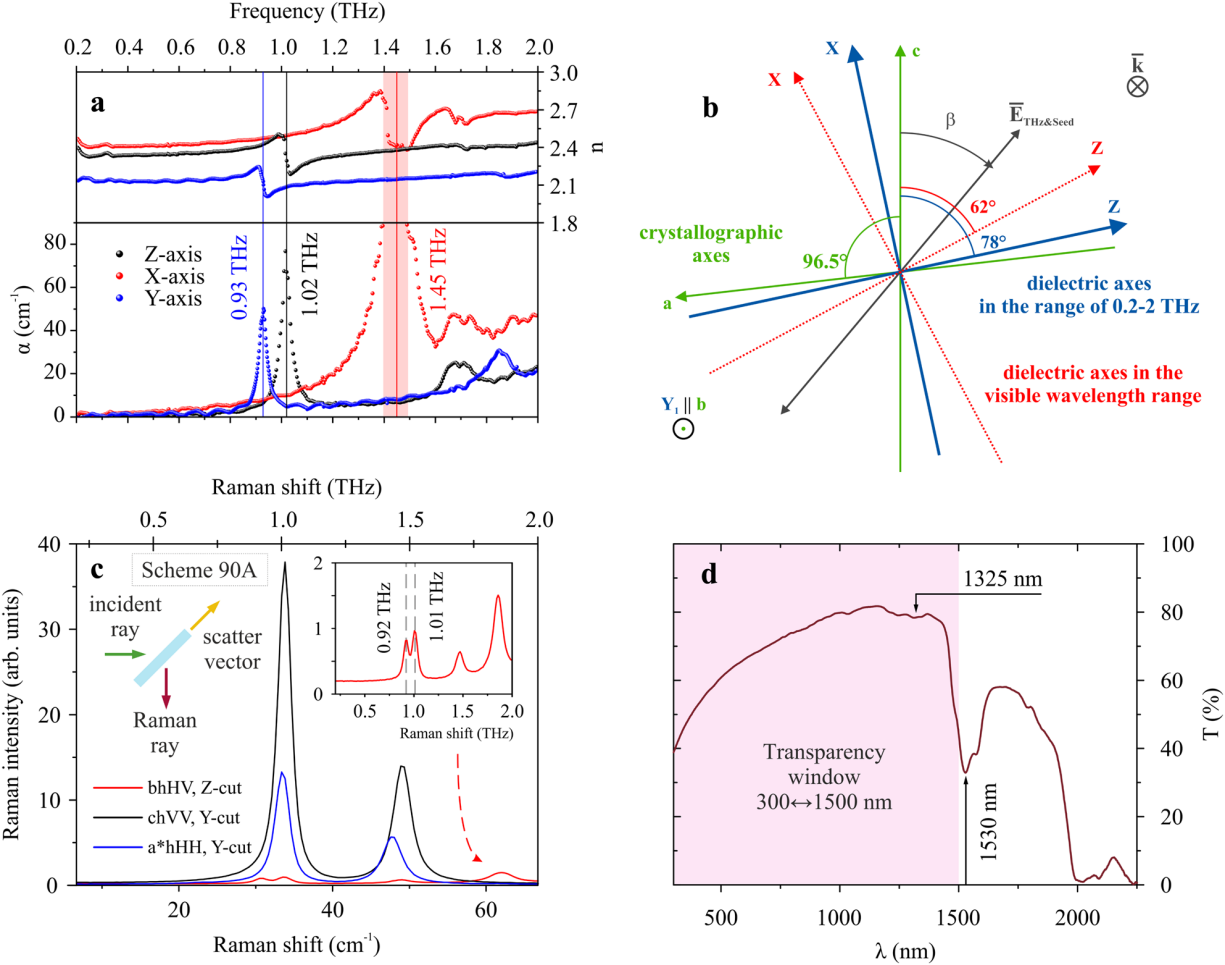
(**a**) Spectra of the refraction and absorption coefficients of GUHP crystal along three principal dielectric axes. The absorption coefficient is presented in terms of field. (**b**) Orientation of the crystallographic and dielectric axes in the Y-cut plane of GUHP crystal. The crystallographic axes are shown in green, the dielectric axes in the visible range are in red, and in the THz range in blue. (**c**) Raman spectra of GUHP crystal for three polarization-axis orientations at 293 K. The angle between the crystallographic axes a and c is $96.5 ^\circ$, so the blue curve corresponds to an orientation orthogonal to the c axis, close to a. The right inset shows an enlarged spectrum of the cross-polarized analyzer and polarizer regime. The left inset represents a schematic diagram of the experiment. (**d**) Transmission spectrum of GUHP crystal in the VIS-NIR spectral range.

Using THz time-domain spectroscopy (THz-TDS), the spectra of the absorption coefficient and refractive index of GUHP crystal were obtained over the frequency range 0.2–2 THz (Fig. 1a). This crystal belongs to the monoclinic syngony^20^; thus, it is birefringent and exhibits three characteristic semi-axes (Fig. 1b). Another feature of such crystals is the coincidence of only one dielectric axis Y with the crystallographic axis b, while axes X and Z are in the plane of the crystallographic axes a and c and generally do not coincide with them. In the visible range GUHP crystal is transparent (absorption coefficient $\alpha < 0.1$ cm$^{-1}$), without any absorption resonance. Therefore, the crystal shows no dispersion along the three dielectric axes, which was confirmed by measurements performed at four different wavelengths, namely 430, 520, 632.8, and 797 nm. At first we characterized the 0.5-mm thick Y-cut sample. In the visible range, the dielectric axis Z differs from the crystallographic axis c by $62^{\circ } \pm 0.5^{\circ }$. In the THz frequency range, this angle, calculated from the TDS-TDS waveforms, is $78 ^{\circ } \pm 1 ^{\circ }$, which is $16 ^{\circ }$ larger than in the visible range. Then the THz absorption spectrum of GUHP crystal was determined along the three dielectric axes. For the X axis, taking into account the significant absorption, it was natural to assume that the peak is located at a frequency $\nu _X=1.45$ THz with a width of no more than $\Delta \nu _X=0.2$ THz. For the Z- and Y- axes, the absorption peaks are located at $\nu _Z=1.02$ and $\nu _Y=0.93$ THz with the line width $\Delta \nu _Z=0.057$ and $\Delta \nu =0.036$ THz, respectively (Fig. 1a). The approximation was carried out using the Lorentzian model. The corresponding Q-factor $Q=\nu /\Delta \nu$ was $Q_X>7.25$, $Q_Z=18\pm 0.4$ and $Q_Y=26.0\pm 0.4$, which significantly exceeds the known absorption peak of the a-axis of the DAST crystal at 1.1 THz: $Q_{a-DAST}=2.8$^22^. In addition, such a resonant character of the interaction of THz radiation with the crystal structure suggests a good efficiency of THz radiation generation in GUHP crystal in the spectral region of resonance peaks.

For a more detailed comprehension of the nature of GUHP crystal’s THz-active phonon modes, we also probed phonons with Raman spectroscopy at 293K, which was a challenging task due to the low frequency of the investigated part of the Raman spectrum. The right-angle Raman scattered light was measured in the 90$^{\circ }$ scheme (see the inset in Fig. 1c). In this figure the Raman spectrum for the Z-cut sample with b crystallographic axis (co-directional with Y dielectric axis) in the scattering plane is shown for horizontal (H) incident polarization and vertical (V) polarization of scattered light (horizontal or vertical with respect to the scattering plane)—HV spectrum. Additionally, VV spectrum for the Y-cut sample with crystallographic axis c in the scattering plane and HH spectrum for the Y-cut sample with a$^*$ axis in the scattering plane are presented in Fig. 1c, where axis a$^*$ is the projection of the crystallographic axis a onto the direction orthogonal to the c axis. It can be seen that the IR active Z- and X-resonances are also strongly Raman active. The Y-mode is weakly excited, and observed only in the case of crossed analyzer and polarizer, and when the crystallographic axis b lies in the scattering plane, co-directed with the dielectric Y axis.

This resonance response of the crystal structure makes GUHP crystal a potential candidate for the role of a THz radiation source with unique spectral characteristics. It has a wide transparency band in the VIS-NIR spectral region, which provides a good efficiency of converting radiation with a frequency from this range to terahertz radiation (Fig. 1d). In addition, the effect of the temperature of the crystal on its spectral properties is of great interest, since the behavior of phonons directly depends on the temperature of the crystal structure. This topic will be discussed in the next subsections. To further investigate the specificity of the observed features of the absorption and refraction coefficients in THz spectra, X-ray diffraction data analysis was performed.

### Crystal structure and bond length temperature dynamics

X-ray diffraction analysis at temperatures 293K and 80K was carried out for the GUHP single crystal (with the best diffraction peak profiles and convergence of intensities of symmetry-equivalent diffraction reflections). The main crystallographic characteristics and parameters of the structure refinement for the GUHP single crystal under study at 293K and 80K are given in Table 1 (full data see in Supplementary materials), and the main interatomic distances are listed in Table 2.

The unit cell of the monoclinic modification of the GUHP single crystal contains 11 crystallographically independent non-hydrogen atoms: one phosphorus atom, two carbon atoms, four nitrogen atoms, four oxygen atoms, and nine hydrogen atoms. Refinement of the structural parameters of the GUHP single crystal at 293K and 80K was performed within the anisotropic approximation of thermal atomic displacements non-hydrogen atoms and in the isotropic displacement approximation thermal parameters of hydrogen atoms within the space group Cc (Z = 4), established previously^19,20^. It should be noted that with decreasing temperature, the unit cell volume of a GUHP single crystal decreases (Table 1).

**Table 1:** Crystallographic characteristics, experimental data, and structure refinement parameters for GUHP.

Chemical formula	$({{ NH}}_2)_2{{ CNHCO}}({{ NH}}_2){{ H}}_2{{ PO}}_3$ ( GUHP)	
Space group, Z	Cc, 4	
Temperature (K)	293	80
a, b, c (${\mathring{A}}$)	6.6982(1), 6.8343(1), 16.3436(2)	6.6828(1), 6.7535(1), 16.2433(1)
$\beta$ (deg)	96.5060(11)	96.5183(8)
V (${\mathring{A}}^3$)	743.351(18)	728.358(12)

**Figure 2:**
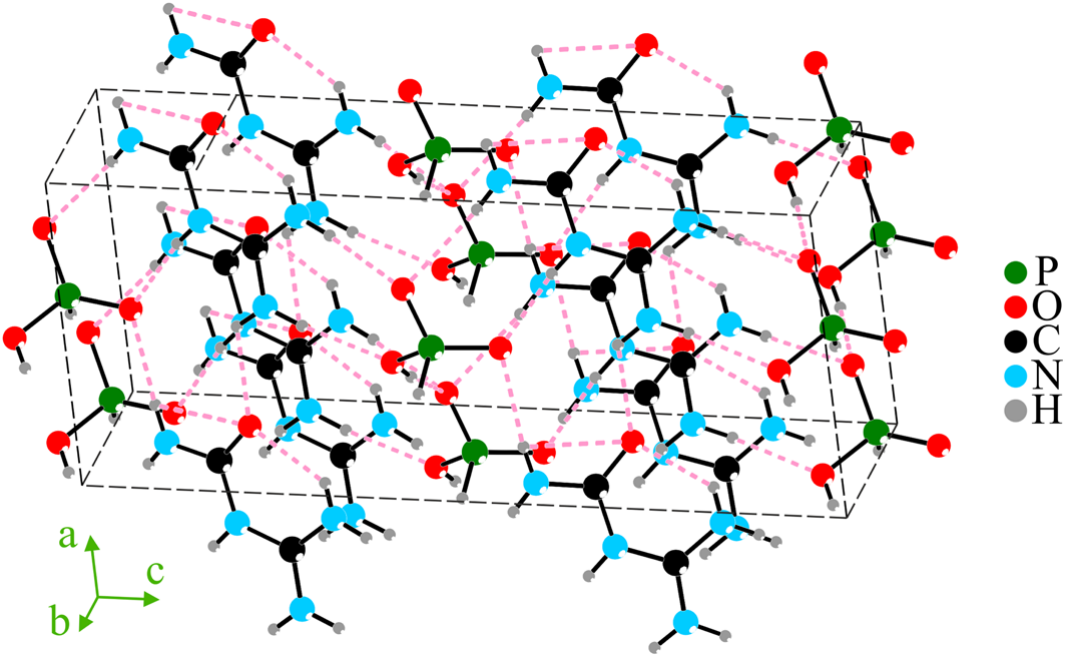
Crystal structure of GUHP crystal. Black solid lines indicate covalent bonds. Pink dashed lines indicate hydrogen bonds. In the diagram, two segregate groups can be distinguished: the organic guanylurea group (accumulations of nitrogen, carbon and oxygen atoms) and the inorganic phosphate group (phosphorus and oxygen atoms).

The GUHP structure (Fig. 2) consists of guanylurea cations (1+) and hydrogen phosphite ions (1−), which form a branched system of fairly weak hydrogen bonds in the GUHP structure (Table 2). Within a guanylurea cation (1+), one hydrogen bond is formed between the oxygen (acceptor) and one of the hydrogen atoms of nitrogen (donor) (Table 2); as a result, the atomic group becomes flat. A similar hydrogen bond is formed between two guanylurea molecules (1+): one oxygen molecule (acceptor) and one hydrogen atom from the nitrogen of another molecule. The presence of a lone-electron pair in the P$^{+3}$ ion explains the formation of a P1_H8 contact with a distance of 1.258(14) ${\mathring{A}}$ at room temperature and 1.304(8) ${\mathring{A}}$ at 80K. In the GUHP structure, this is the shortest contact for the P1 atom, since the bonds with oxygen at the P1 atom are longer (about 1.5 ${\mathring{A}}$) (Table 2). And for the H8 atom this is the only contact, since the H8 atom does not participate in the formation of any hydrogen bonds in the GUHP structure. The H9 atom, for which the O2-P1 atom is the donor, forms a hydrogen bond with the O3_P1 atom of the neighboring H$_2$PO$_3^-$ ion. With the nitrogen atoms of the guanylurea ion (1+), the oxygen atoms of the hydrophosphite ion form rather weak contacts with a length from 2.77 to 3.07 ${\mathring{A}}$ (Table 2). Thus, the hydrogenphosphite ion in the GUHP structure is quite independent. At the stage of refining coordinates of P, N, O and C atoms and their thermal parameters within the anisotropic approximation, we refined the occupancies of P atom (the heaviest atom in the structure) of intrinsic crystallographic sites. The occupancy of the position of the P1 atom at room temperature was 0.987(2), and at 80K–0.992(1). The uninterpretable peaks were revealed in the difference electron-density maps near P and O atomic sites. Therefore, it is possible that P atoms and surrounding it oxygen atoms are structurally disordered over several positions. However, it does not seem possible to refine the P and O atomic distributions over several close positions because of the strong correlation between the structural parameters.

**Table 2:** Interatomic distances in the structures of $({{ NH}}_2)_2{{ CNHCO}}({{ NH}}_2){{ H}}_2{{ PO}}_3$ single crystal at 293K and 80K.

Chemical bond	293K		80K	
	Distances (${\mathring{A}}$)			
P1–O1_P1	1.4939 (6)		1.4985 (3)	
-O2_P1	1.5777 (6)		1.5868 (3)	
-O3_P1	1.5060 (5)		1.5134 (2)	
-H8	1.258 (14)		1.304 (8)	
C1 -O1	1.2275 (7)		1.2357 (3)	
-N1	1.3333 (9)		1.3363 (4)	
-N2	1.3930 (6)		1.3945 (3)	
C2 -N2	1.3546 (8)		1.3587 (4)	
-N3	1.3168 (7)		1.3224 (4)	
-N4	1.3197 (6)		1.3237 (3)	
Hydrogen bonds				
D-H.....A	T (K)	D-H distances (${\mathring{A}}$)	H......A distances (${\mathring{A}}$)	D-A distances (${\mathring{A}}$)
	293	0.817 (17)	1.783 (16)	2.5872 (8)
O2_P1-H9... O3_P1	80	0.810 (10)	1.767 (9)	2.5736 (3)
	293	0.905 (14)	2.126 (14)	3.0286 (7)
N1-H5_N1... O1_P1	80	0.848 (12)	2.172 (11)	3.0063 (3)
	293	0.763 (15)	2.319 (16)	3.0743 (7)
N1-H6_N1....O3_P1	80	0.842 (9)	2.201 (9)	3.0348 (3)
	293	0.843 (11)	1.944 (11)	2.7710 (6)
N2-H7_N2... O1_P1	80	0.893 (9)	1.885 (9)	2.7701 (3)
	293	0.811 (12)	2.042 (13)	2.6409 (8)
N3-H3_N3......O1	80	0.904 (9)	1.940 (10)	2.6399 (4)
	293	0.810 (15)	2.133 (15)	2.9378 (8)
N3-H4_N3....O3_P1	80	0.939 (10)	2.002 (10)	2.9409 (4)
	293	0.796 (15)	2.252 (16)	2.7080 (6)
N4-H1_N4.......O1	80	0.804 (8)	2.128 (8)	2.6938 (3)
	293	0.856 (16)	2.099 (16)	2.9453 (10)
N4-H2_N4....O2_P1	80	0.786 (11)	2.146 (11)	2.9215 (5)

It should be noted that with decreasing temperature, the unit cell volume of a GUHP single crystal decreases, the values of the parameters of thermal displacements of all atoms decrease, no significant changes in the geometric parameters of the guanylurea molecule (1+) were revealed, but a redistribution of the electron density in the hydrophosphite ion was established. The obtained information about the investigated structures was deposited with the Cambridge Crystallographic Data Center (CCDC nos. 2055448, 2055458). The data obtained can be used to simulate the crystal vibrations that we observe in THz spectra of the absorption and refractive coefficients. It is also interesting to predict the low-temperature dynamics of this phenomenon. To further investigate the THz absorption spectrum, the transmittance properties of GUHP crystal were measured at various temperatures and performed density functional theory (DFT) simulations from the obtained X-ray diffraction analysis’ data.

### Computational simulations, temperature dynamics and THz generation

**Figure 3:**
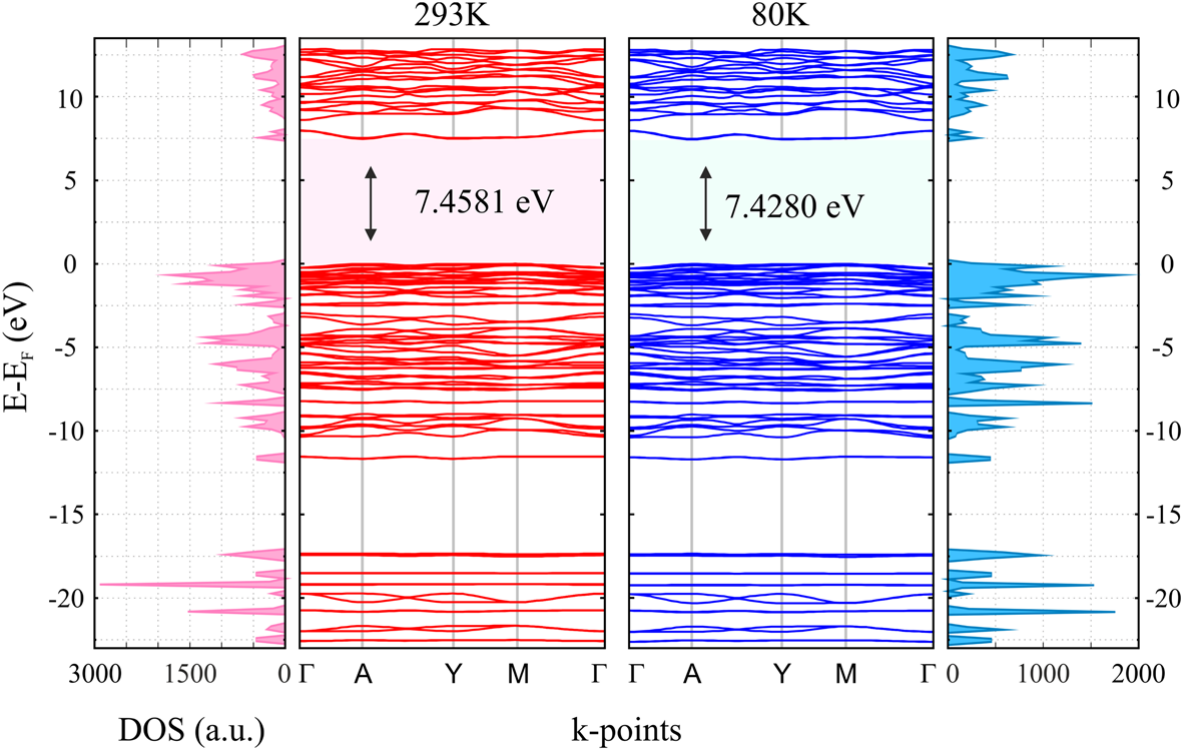
Calculated band structure and density of states (DOS) for GUHP. The energy of the band gap for both temperatures corresponds to the wavelength of the VUV spectral region, which is 166.24 nm (293K) and 166.91 nm (80K).

For a comprehensive study of dielectric properties of GUHP crystal, the band structure of the electronic subsystem was simulated. The calculated values of the indirect band gap are 7.4581 eV ($E_F=-7.7291$ eV) and 7.4280 eV ($E_F=-7.6886$ eV) corresponding to the experimental lattice parameters at 293K and 80K, respectively. The resulting band structures are illustrated in Fig. 3. It is obvious now, that at least VUV radiation is required for the effective excitation of the crystal electronic subsystem. This leads to the conclusion that it is the phonon subsystem that will play a decisive role in the nonlinear optical processes in GUHP crystal, since it is extremely unstable to ultraviolet radiation being organic structure saturated with a large number of hydrogen bonds.

With the aim of detailed study of the phonon dynamics in GUHP crystal and the subsequent simulation of the generated radiation, the spectral properties of the THz radiation transparency of the sample at cryogenic temperatures were investigated. Experiments were carried out on the Y-cut sample 0.5 mm thick. To characterize the effect of the temperature on the dielectric properties of GUHP crystal, the absorption and refraction spectra were obtained in direction of the Z dielectric axis. Due to the fact that the wide electronic band gap of GUHP crystal practically does not allow the appearance of the free charge carriers in the bulk of the crystal, the experimental results were simulated using the standard model of dialectic permeability in structural resonance media. The dielectric phonon absorption resonances were fitted with the Lorentzian expressions for complex dielectric permittivity:

1 $$\begin{aligned} \widetilde{\varepsilon }(v)=\varepsilon _{\infty }+\frac{v_{p h}^{2}\left( \varepsilon _{0}-\varepsilon _{\infty }\right) }{v_{p h}^{2}-v^{2}-i v \hat{\gamma }_{p h}}=\varepsilon ^{\prime }(v)+i \varepsilon ^{\prime \prime }(v) \end{aligned}$$

where in Eq. (1) *v* is the frequency of THz radiation, $\varepsilon _{\infty }$ and $\varepsilon _{0}$ are dielectric constants in the high- and low-frequency limits, respectively; $\nu _{p h}$ is the central resonance frequency, $\hat{\gamma }_{p h}=\frac{\gamma _{p h}}{2 \pi }$ is the reduced damping parameter of the structure vibrations. Then the real and imaginary parts of the dielectric constant and their relationship with the absorption and refractive indices are expressed as:

2 $$\begin{aligned}&\widetilde{\varepsilon }(v)=\widetilde{n}(v)^{2}=\left( n(v)+i \kappa (\nu )\right) ^{2} \end{aligned}$$

3 $$\begin{aligned}&\varepsilon ^{\prime }(v)=\varepsilon _{\infty }+\frac{v_{p h}^{2}\left( \varepsilon _{0}-\varepsilon _{\infty }\right) \left( v_{p h}^{2}-v^{2}\right) }{\left( v_{p h}^{2}-v^{2}\right) ^{2}+\left( v \hat{\gamma }_{p h}\right) ^{2}}=n(v)^{2}-\kappa (\nu )^{2} \end{aligned}$$

4 $$\begin{aligned}&\varepsilon ^{\prime \prime }(v)=\frac{v_{p h}^{2}\left( \varepsilon _{0}-\varepsilon _{\infty }\right) v \hat{\gamma }_{p h}}{\left( v_{p h}^{2}-v^{2}\right) ^{2}+\left( v \hat{\gamma }_{p h}\right) ^{2}}=2 n(v) \kappa (\nu ) \end{aligned}$$

where *n* is the refractive index; *c* is the speed of light in vacuum; $\kappa (\nu )=\frac{c \alpha (v)}{2 \pi v}$ is the extinction coefficient and $\alpha (\nu )$ is the field absorption coefficient.

Since the complex refractive index was obtained experimentally, it is necessary to express its components. It is also necessary to take into account the background part of the experimental data using the usual additional term similar to Sellmeier equation with $a_{i}$ and $b_{j}$ parameters.

5 $$\begin{aligned} n(v)= & {} \sqrt{\frac{1}{2} \varepsilon ^{\prime }(v)\left[ \sqrt{1+\left( \frac{\varepsilon ^{\prime \prime }(v)}{\varepsilon ^{\prime }(v)}\right) ^{2}}+1\right] +a_{1}+\frac{a_{2}}{1-a_{3} v^{2} / c^{2}}} \end{aligned}$$

6 $$\begin{aligned} \alpha (v)= & {} \frac{2 \pi v}{c} \sqrt{\frac{1}{2} \varepsilon ^{\prime }(v)\left[ \sqrt{1+\left( \frac{\varepsilon ^{\prime \prime }(v)}{\varepsilon ^{\prime }(v)}\right) ^{2}}-1\right] +b_{1}+\frac{b_{2}}{1-b_{3} v^{2} / c^{2}}} \end{aligned}$$

**Figure 4:**
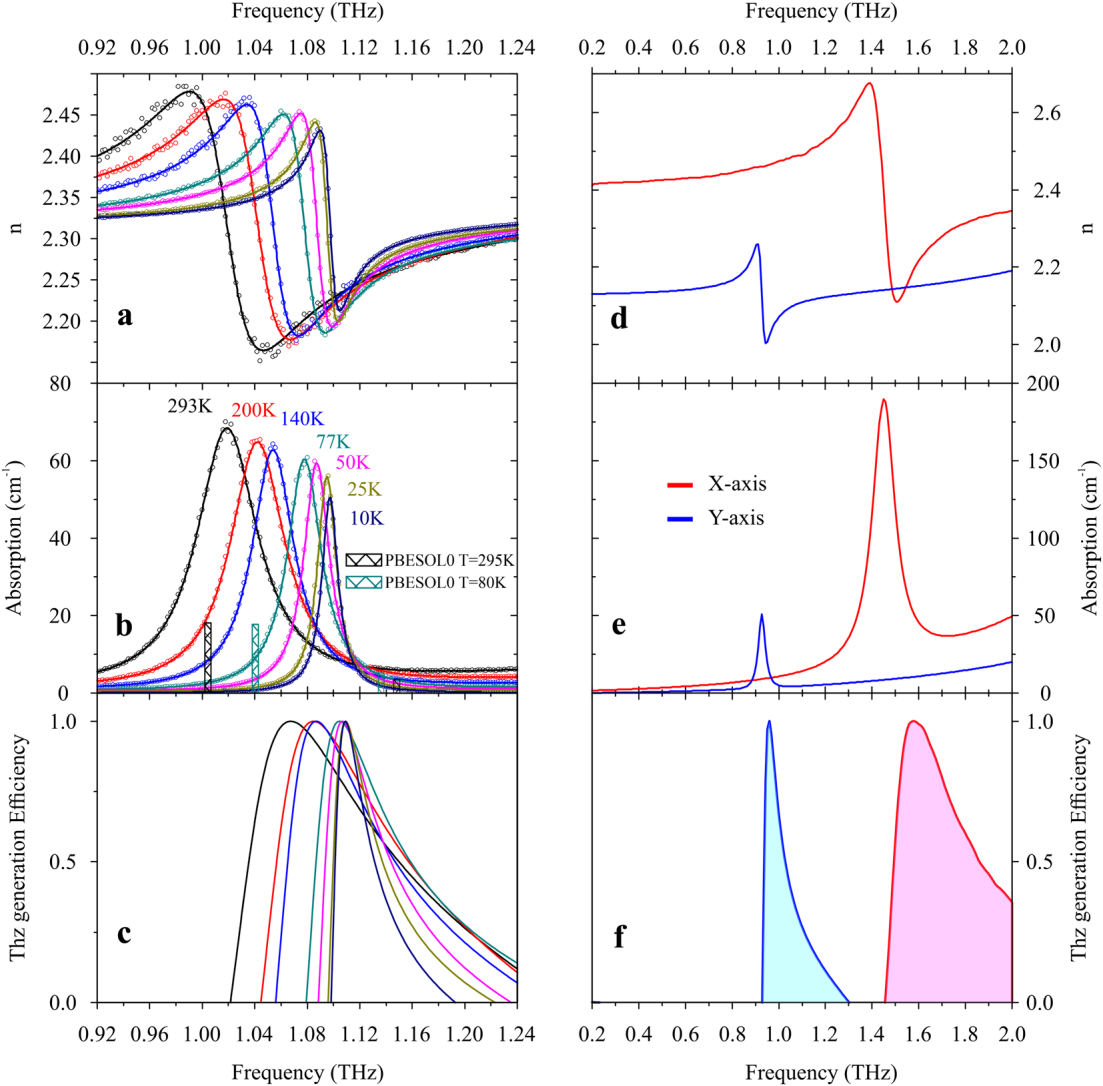
(**a**,**b**) THz spectra of the absorption and refraction coefficients for Z-mode at different temperatures. Dots represent experimental data. Solid curves indicate the results of the modeling. The columns correspond to the results of the DFT simulation. (**c**) Simulated normalized generation spectra of terahertz radiation in GUHP crystal for the Z axis at different temperatures. (**d**,**e**) THz spectra of the absorption and refraction coefficients for the X and Y axes at room temperature. The experimental data presented earlier was approximated by the chosen phonon-resonance model for dielectric permittivity. (**f**) Simulated normalized generation spectra of terahertz radiation in GUHP crystal along X and Y axes at room temperature.

The spectral dependencies of the absorption and refraction coefficients of GUHP crystal have been experimentally determined in the THz frequency range along the selected direction Z at various temperatures: from room temperature to cryogenic one. Dielectric properties of GUHP crystal were obtained from these dependences on the basis of the classical damped harmonic oscillator model for the phonon-resonance structure^23^. Using the parameters of the medium determined in this way, in the future it will be possible to describe the properties of THz radiation generated in the crystal under study by nonlinear optical methods. Figure 4a,b show the refraction and absorption spectra of GUHP crystal when the polarization of the terahertz wave is oriented along the Z direction. Figure 4d,e represent these parameters for X- and Y-axes.

As a result of approximation of the experimental data by phonons model formulas, the dielectric parameters of GUHP crystal at 293K were determined for the $0.2-2$ THz spectral range for 3 principal directions. At constant electric field and 293K, the dielectric constants and the refractive indices: $\varepsilon _{0Y}=4.54$ and $n_Y (0)=2.13$ for Y-axis, $\varepsilon _{0Z}=5.33$ and $n_Z (0)=2.31$ for Z-axis, $\varepsilon _{0X}=5.79$ and $n_X (0)=2.41$ for X-axis. The dependences of the position and FWHM of the absorption peak were investigated as a function of temperature. Both of these parameters change linearly with the cooling of the crystal lattice. The total shift in the position of the absorption peak with a change in temperature from 293 to 10K is 79 GHz. In this case, the width of the peak decreases to $\Delta \varepsilon _Z=15$ GHz, and the temperature dynamics lead to a hyperbolic increase in the oscillator strength up to $Q_Z=72$. Such a change in the spectral characteristics of GUHP crystal in the THz frequency range leads to the sharpening of the spectral region of phase matching for the nonlinear optical process of generating THz radiation by optical rectification. We also predict an increase in the nonlinear optical conversion efficiency due to a decrease in damping processes in the crystal lattice at low temperatures.

Using DFT modeling, the low-frequency vibrational modes (below 2 THz) and its IR intensities were identified. They are presented in Table 3 as well as the experimental results for IR absorption and Raman lines.

**Table 3:** Low-frequency vibrational modes of GUHP crystal unit cell at T = 80K and T = 293K.

T = 293K				T = 80K				Irreducible representation
Freq. (THz)			Calc. IR intensity (km/mol)	Freq. (THz)			Calc. IR intensity (km/mol)	
Calc.	Exp.	Raman		Calc.	Exp.	Raman		
None	0.928	0.92	None	None	Not meas.	Not meas.	None	Interlayer
1.004	1.019	1.01	0.78	1.040	1.078	Not meas.	0.76	A’
1.149	None	None	0.15	1.136	None	None	0.22	B”
1.484	1.45	1.45	2.78	1.549	Not meas.	Not meas.	3.47	A’

As one can see from the absorption spectra (Fig. 4b) the simulated vibrations match with the experimental data. The spectra of the absorption coefficient clearly show a blue shift of the corresponding resonances upon cooling the crystal. In the experiment, when GUHP crystal was cooled from room temperature to T = 80K, the absorption peak for the Z-axis shifted by 59 GHz, while the model predicts a shift of 36 GHz. This typical pattern of the absorption peaks behavior indicates that THz phonons in GUHP crystal are temperature-dependent, which is confirmed by the modern model concepts^10–12^. If you look at Table 2, you can see that when the crystal structure of GUHP is cooled, the lengths of intraionic covalent bonds increase, while the lengths of hydrogen bonds (more precisely, the distances between donors and acceptors participating in hydrogen bonds) decrease. The nature of this phenomenon will be considered in more detail in subsequent works. However, it can already be stated that, since upon cooling the crystal, we observe a shift of the absorption peaks to the high-frequency region, the bonds responsible for the oscillations excited by THz radiation should be shortened upon cooling. Hence, it follows that it is the hydrogen bonds that are responsible for THz absorption in GUHP crystal.

## Discussion

**Figure 5:**
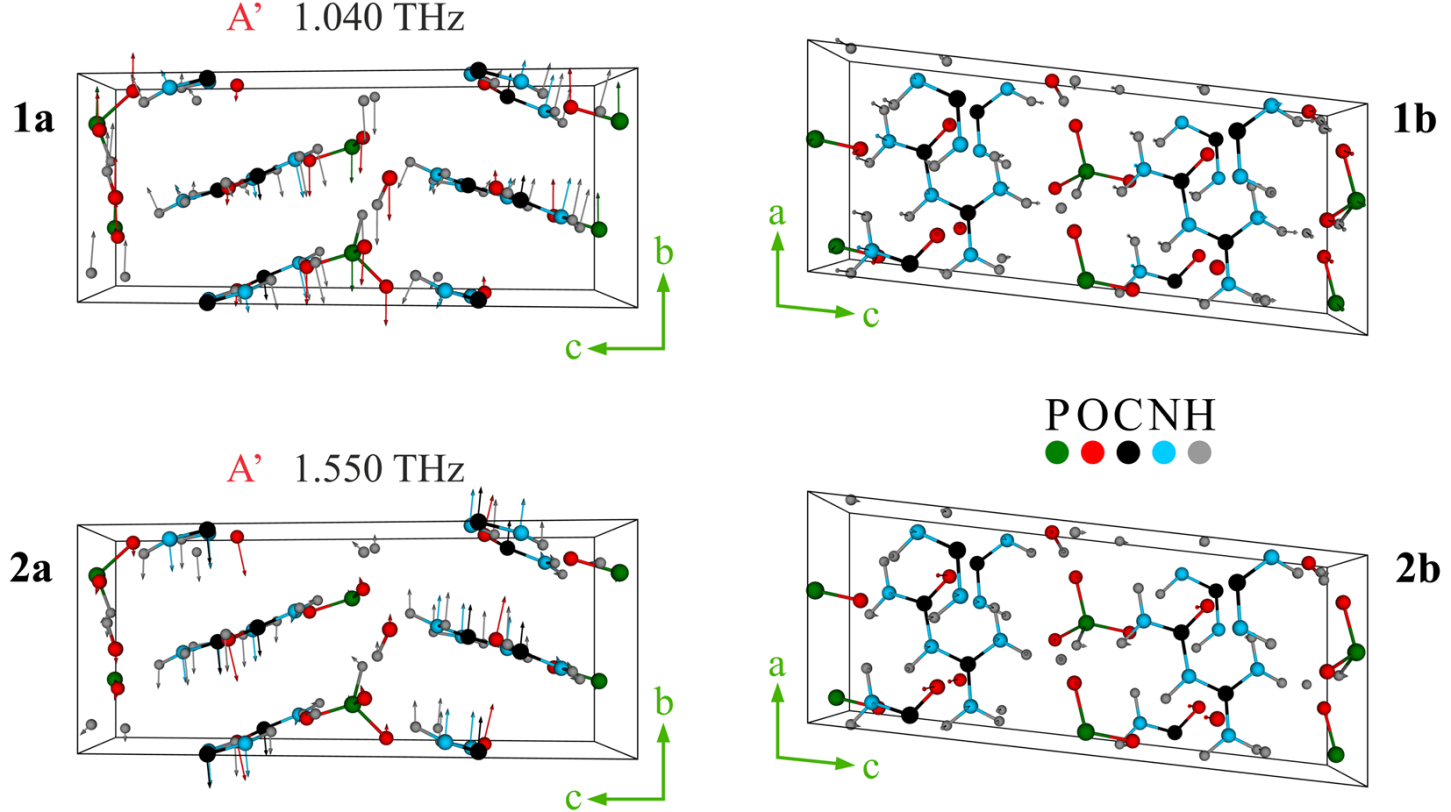
Visualization of the low-frequency vibrational modes of A’ symmetry of GUHP crystal at 80K. The axes indicate crystallographic axes *abc*. 1*a*, 1*b* represent 1.040 THz mode for *a* and *b* cut crystal planes respectively. 2*a*, 2*b* represent 1.550 THz mode for the *a* and *b* cut crystal planes respectively.

Figure 5 illustrates vibrational modes of A’ symmetry of the GUPH crystal unit cell in two projections according to a-axis (left) and b-axis (right) obtained for experimental lattice parameters at T = 80K (Table 2). These vibrational modes can be characterized as translational and librational motions of whole molecular fragments in the unit cell in different directions. Vibrations of the structure at T = 293K are the same.

The data obtained as a result of computer simulation within the framework of the selected DFT modelling show no resonance corresponding to the absorption peak of Y (0.93 THz at 293K). It could be due to the limitations of the chosen model that does not take into account the pronounced layered structure of GUHP crystal, which is not a “classic” 2D structure built by flat atomic layers. However, quasi-two-dimensional layers formed by semi-organic molecules of guanylphosphiteurea in the form of a herringbone are clearly visible (Fig. 5). Another feature of layered crystals is the appearance of the so-called interlayer breathing and shear phonon modes, which are associated with the complex relative motion of entire layers. There are numerous examples of such structures described in the literature (graphite and graphene^24,25^; MoS$_2$ and WSe$_2$^26^). However, such modes have not yet been observed for complex molecular crystals. We assume that the Raman and IR active modes at 0.93 THz correspond precisely to interlayer vibrational oscillations, which are expressed in the collective movement of layers towards each other (breathing) or along each other (shearing). Since the layers in GUHP crystal are hydrogen bonded, interlayer phonon modes also describe hydrogen bonded vibrations.

Based on the obtained THz spectra of the absorption and refraction coefficients of GUHP crystal we assumed that in the spectral region of the right slope of the absorption peak (in each principal direction X, Y, Z) the generation of THz radiation could be efficient due to the anomalous dispersion. In order to predict the spectral properties of THz radiation generated in GUHP crystal due to the difference frequency generation, we used the experimental data obtained for the spectra of transmission of the crystal under study at different orientations and temperatures. For the simulation of spectral features of THz radiation generated in GUHP crystal the classical nonlinear optical formalism was used^27^. In the constant and plane wave approximation, the equation for THz field amplitude can be presented as follows:

7 $$\begin{aligned} {\mathcal {E}}_{T H z}(\Omega )= \frac{i 2 \pi \Omega ^{2} \chi _{e f f}^{(2)}(\Omega ) {\mathcal {E}}\left( \omega _{1}\right) {\mathcal {E}}^{*}\left( \omega _{2}\right) \left[ e^{i\left( k_{1}-k_{2}-k_{T H z}\right) L}-e^{-\beta _{T H z} L}\right] }{c^{2} \widetilde{k}_{T H z}\left[ i\left( k_{1}-k_{2}-k_{T H z}\right) +\beta _{T H z}\right] } \end{aligned}$$

In Eq. (7) we suppose that all waves propagate along the $\varvec{L}$ direction; $\widetilde{k}_{T H z}=k_{T H z}+i \beta _{T H z}$ and $\beta _{THz}=\alpha _{THz}=\Omega \kappa _{THz}/c$, where all values depend on the THz frequency $\Omega =2\pi \nu$.

Since GUHP crystal has a transparency window in the visible and NIR spectral regions, the absorption coefficients for the pump waves are omitted in the equation. We distinguish the noncritical phase matching as one of the main factor that determines the THz generation spectrum profile. This is defined by the simplified equation in the case of approached phase matching $\left( k_{1}-k_{2}-k_{T H z}\right) {\rightarrow 0}$:

8 $$\begin{aligned} \left| {\mathcal {E}}_{T H z}(\Omega )\right| \sim \frac{\left| \chi _{\text {eff }}^{(2)}\right| (1-e^{- \kappa _{T H z} \Omega L /c})}{\kappa _{T H z} \sqrt{n_{T H z}^{2}+\kappa _{T H z}^{2}}} \end{aligned}$$

Since the refractive index in the THz region has anomalous dispersion on the right slope of the absorption peak, a similar denominator in the expression above will show an efficiency peak in the same region. In addition to the phase matching factor, the enhancement of the second-order nonlinear susceptibility in the spectral region of the resonance can plays a significant role in the generation of THz radiation at phonon resonance. In^9^ it was predicted for some crystal symmetry classes that $\chi ^{(2)}(\Omega )$ dispersion does not follow the linear susceptibility dispersion and has a maximum value on the right slope of the phonon absorption peak. This theory is of interest and will be the subject of future studies. We also predict that due to the resonance character of the THz dielectric properties, the weak absorption and dispersion of the refractive index in the VIS and NIR spectral region (500–1500 nm), nonlinear THz generation in GUHP crystal should not depend on the pump laser wavelength, which would mean noncritical phase matching. The results of modeling the spectrum of the THz radiation generation in GUHP crystal for three principal orientations at different temperatures are presented in Fig. 4c, f. Experimental evidence is needed to establish the veracity of the above presented model. We used two THz-TDS spectrometers described in the experimental scheme analogous to the one described below.

The next step was experimental verification of the simulated THz radiation spectra. For this purpose, a 500 $\mu$m thick Y-cut sample of GUHP crystal was prepared. It served as a source of THz radiation in a standard THz time-domain experimental scheme. We chose an excitation wavelengths of 1325 and 797 nm, which falls within the transparency window. The crystal was excited by a pump wave polarized along the Z or X THz dielectric axis. We collected the THz radiation along the same axis as the initial laser radiation with the help of a wire-grid polarizer.

**Figure 6:**
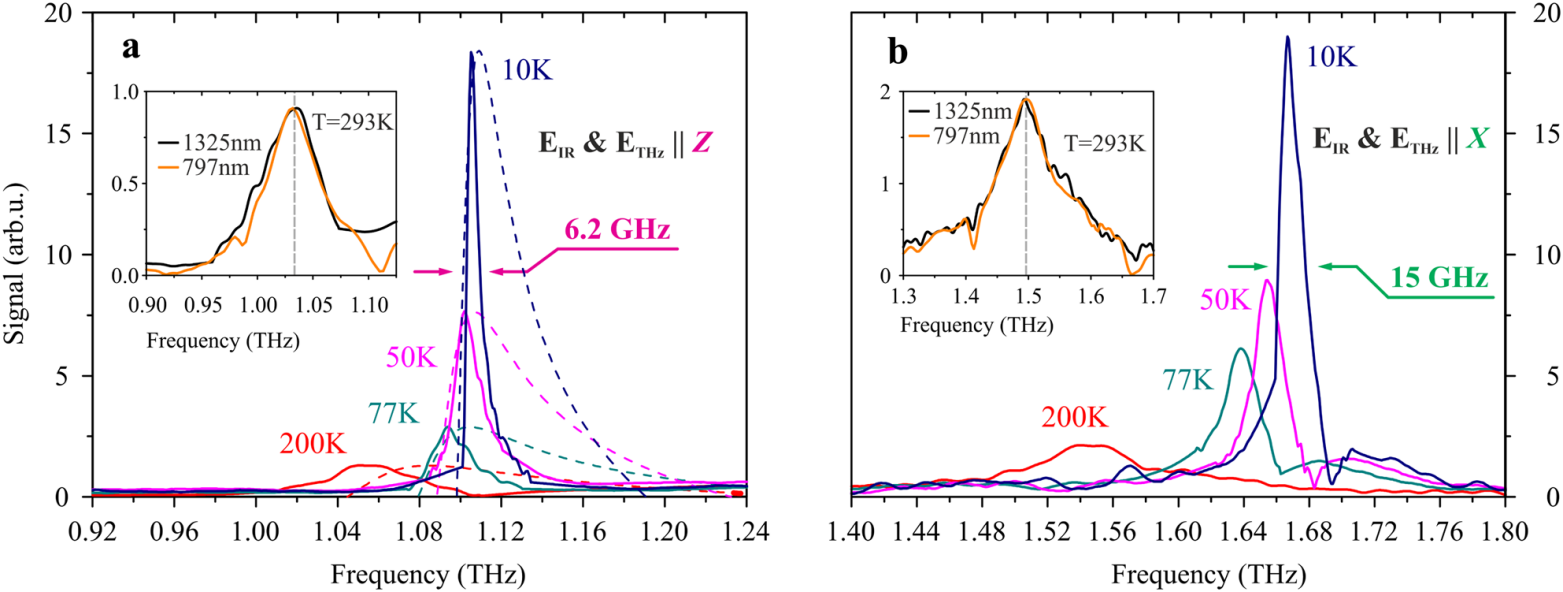
THz field spectra at Z (**a**) and X (**b**) mode orientation at different temperatures. The pump wavelength is 1325 nm. Solid lines represent experimental data; dashed lines represent phase matching approximations using experimental transmission spectra data. The insets represent the comparison of THz radiation spectra generated at 293K in the case of two pump laser wavelengths: 1325 nm and 797 nm.

Figure 6a displays the spectra of THz radiation in the case of Z mode detection (IR pump and THz electric field polarized along Z-axis). All spectra were normalized at the peak level of the Z-mode peak at room temperature. At the same time, THz radiation was also generated along the X axis but with different spectral properties related to the transparency spectral properties discussed earlier. Figure 6b displays the spectra of THz radiation in the case of X mode detection (IR pump and THz electric field polarized along X-axis). While temperature changes from room to cryogenic, the damping of the electric field oscillations weakens, which is clearly expressed in the spectral representation as emphasizing and narrowing of the generation frequency peak. The energy of THz pulses reached 700 pJ at low temperatures, which corresponds to the efficiency of $8\cdot 10^{-7}$. By this parameter, GUHP crystal is inferior to the widespread broadband THz radiation sources: for example, for ZnTe crystal the conversion coefficient can reach^28^ about 10$^{-5}$. In the case of GUHP crystal, the phase matching turned out to be frequency independent: the nonlinear conversion process was comparable at wavelengths of 1325 nm and 797 nm (insets in Fig. 6). It is also obvious that the spectral densities in the main GUHP generation frequency bands are higher than that for broadband THz sources, such as ZnTe crystals. In addition, in comparison with other known crystals with a narrow lasing band (${{ BaGa}}_4{{ Se}}_7$ crystal^16^ efficiency is  10$^{-8}$), GUHP crystal has a higher conversion efficiency. And finally GUHP crystal has a high optical breakdown threshold, which is extremely important for nonlinear optics applications.

The simulation of THz radiation spectra are in a good agreement with the experimentally obtained results. This fact strictly confirms our hypothesis that the spectral properties of the THz radiation generated in GUHP crystal are mainly determined by the dielectric response associated with the phonon subsystem of the lattice. We have determined that GUHP crystal is an effective nonlinear media for THz radiation generation with tightly tunable spectral properties, which can be controlled both by the orientation and the temperature of the crystal. Based on the above, we can say that GUHP crystal is extremely important for THz applications.

## Methods

### Materials and sample preparation

The synthesis of GUHP solutions was carried out by dissolving dicyandiamide (${{ HNC}}({{ NH}}_2){{ NHCN}}$ or ${{ C}}_2{{ H}}_4{{ N}}_4$) 99.5$\%$ and phosphorous acid $({{ H}}_3{{PO}}_3)$ 98$\%$ in distilled water:

$$\begin{aligned} C_2H_4N_4 + H_3PO_4 + H_2O \rightleftharpoons (NH_2)_2CNHCO(NH_2)H_2PO_3 \end{aligned}$$

The crystals were grown by isothermal evaporation of the solvent at 48 $^{\circ }$C. (001) plates with dimensions of $3\times 3\times 3$ mm$^3$ were used as seed crystals.

### Terahertz time-domain spectrometers (THz-TDS) at different temperatures and various excitation wavelengths

We used THz time-domain spectrometer for acquiring the THz-TDS measurements and to study of dielectric properties of GUHP. THz-TDS spectrometer uses 797 nm pulses of Ti:sapphire femtosecond regenerative amplifier (Spectra Physics Spitfire Pro) at 1 kHz repetition rate and 2.2 W output power with the pulse duration 120 fs. An electro-optical sampling with a 1 mm thick $\langle 110\rangle$ ZnTe crystal was used as a detector of THz pulses. The frequency range of the TDS spectrometer is 0.2 to 2.5 THz. A two-color optical breakdown plasma in air was used as a source of THz radiation. A nonlinear $\beta$-BBO crystal 100 $\mu$m thickness was used as the second harmonic source. The THz radiation power reached 10 $\mu$W. Cooling of the sample was carried out using a closed-cycle cryostat (SHI Cryogenics Group) capable of cooling the sample to cryogenic temperatures. Silicon diode temperature sensors (Lake Shore Cryotronics, Inc.) were used to determine the temperature of the copper sample holder. To reduce the attenuation of the THz signal by water vapor, the path of the THz pulse has been laid in a chamber filled with nitrogen gas.

To study the THz generation properties of GUHP the CDP2017 femtosecond optical parametric amplifier was used as a wavelength-tunable (1.1–1.6 $\mu$m) fs IR pulse source pumped by the Spectra Physics Spitfire Pro Ti:sapphire regenerative-amplifier system that delivered optical pump pulses of 120-fs duration, of 797-nm central wavelength and also was used as a laser source for the crystal pumping. The experimental setup schemes can be found in the Supplementary section.

### X-ray diffraction analysis

X-ray intensity data sets for single crystals of no more than 0.5 mm in size were collected at room temperature and at 80K on X-ray diffractometer XtaLAB Synergy R, DW system, HyPix-Arc 150 Hybrid Pixel Array Detector (Rigaku Oxford Diffraction, Abingdon, Oxfordshire, UK) (MoK$\alpha$ radiation). Integration of peaks, LP correction and sample shape absorption correction were performed using a program entering the mathematical package of CrysAlis CCD diffractometers^29^. All other crystallographic calculations (introduction of anomalous scattering corrections, and averaging of symmetry-equivalent reflections) were performed using the Jana2006 software. The model of the atomic crystal structure was obtained by the Charge flipping method using the SUPERFLIP program from the Jana2006 software package^30^. The structural parameters were refined using the least squares method in the full-matrix version.

### Raman spectroscopy

Raman experiment were carried out with a TriVista777 spectrometer in a three-grating mode. Raman scattering was excited by radiation from a solid-state laser with a wavelength of 532 nm, whose parasitic background was suppressed by a monochromator^31,32^. The entrance slit was 50 $\mu$m (1 cm$^{-1}$ of the FWHM spectral resolution). Wavelength calibration of the spectrometer was done with emission spectrum of a neon-discharge lamp. Raman scattering spectrum was recorded for the Raman shift from 5 to 500 cm$^{-1}$.

### Density functional theory calculations

Solid-state DFT simulations were performed using the fully-periodic CRYSTAL17 software package^33^. We use the PBESOL0 hybrid functional^34^ (with 25% of Hartree-Fock exchange mixing), and an all-electron triple-$\zeta$ valence polarization (pob-TZVP) basis set^35^. A total energy convergence threshold of 10$^{-11}$ au and truncation criteria for bielectronic integrals of 10, 10, 10, 10 and 20 are used. 494,123 grid points are used in calculations. A pruned (75, 974) grid is used, having 75 radial points and a maximum number of 974 angular points on the Lebedev surface in regions relevant for chemical bonding. Each atomic grid is split into five shells with different angular grids. The Monkhorst–Pack scheme^36^ with $8\times 8\times 8$ k-point mesh is used for sampling of the Brillouin zone. London dispersion forces were accounted for using the Grimme DFT-D3 correction^37^, with the Becke–Johnson damping (BJ)^38^ (s$_6$=1.000, a$_1$=0.4466, s$_8$=2.9491, a$_2$=6.1742) and Axildor–Teller–Muto three-body dispersion correction (ATM). To correct for the basis set superposition error (BSSE) present in GTO calculations, we used the geometrical counterpoise (gCP) correction with automatic parameter setup^39^.

In the geometry optimization, the lattice parameters are held at the experimental values at 80K and 293K, and the crystallographic symmetry elements are preserved, namely, monoclinic unit cell with space group Cc and parameters a=6.6794 ${\mathring{A}}$, b=6.7532 ${\mathring{A}}$, c=16.2342 ${\mathring{A}}$, and $\beta$=96.514$^\circ$ (80K); a=6.6905 ${\mathring{A}}$, b=6.8283 ${\mathring{A}}$, c=16.3312 ${\mathring{A}}$, and $\beta$=96.510$^\circ$ (293K). Coordinates of all atoms are optimized using the following criteria: RMS of gradient, 10$^{-6}$ au; RMS of estimated displacements, $5\cdot 10^{-6}$ au; and threshold of the energy change between optimization steps, 10$^{-11}$ au. The optimized coordinates can be found in Supplementary materials.

The band structure and density of states (DOS) calculations are performed after geometry optimization. The correct prediction of the band gap is a matter of debate in DFT. Here we consider the band gap within the Kohn–Sham formalism as the difference between the top of the valence bands and the bottom of the conduction bands. Since semilocal functionals underestimate the band gap, the use of hybrid functionals designed for solids such as PBESOL0 can reduce the error^40^. The calculation of the vibrational frequencies at the $\Gamma$ point is performed within the harmonic approximation by numerical evaluation of the Hessian matrix elements as the first derivatives of the atomic energy gradients using central-difference formula with the displacement of each atom along the Cartesian coordinates by 0.001 ${\mathring{A}}$ in two directions^41^. The IR intensities are calculated with the aid of coupled-perturbed Hartree–Fock/Kohn–Sham approach^42^.

## Supplementary information

Supplementary Information.
