# Author Correction: A monoclinic semiorganic molecular crystal GUHP for terahertz photonics and optoelectronics

**DOI:** 10.1038/s41598-022-07362-6

**Published:** 2022-02-24

**Authors:** Anton Sinko, Peter Solyankin, Aleksey Kargovsky, Vera Manomenova, Elena Rudneva, Natalia Kozlova, Natalia Sorokina, Fedor Minakov, Sergei Kuznetsov, Nazar Nikolaev, Nikolay Surovtsev, Ilya Ozheredov, Alexey Voloshin, Alexander Shkurinov

**Affiliations:** 1 Faculty of Physics, Lomonosov Moscow State University, GSP‑1, 1‑2 Leninskiye Gory, Moscow, Russia 119991; 2 Institute on Laser and Information Technologies - Branch of the Federal Scientific Research Center “Crystallography and Photonics” of the Russian Academy of Sciences, Svyatoozerskaya Str. 1, Shatura, Russia 140700; 3 Penza State University, Krasnaya Str. 40, Penza, Russia 440026; 4 Shubnikov Institute of Crystallography of the Federal Scientific Research Center “Crystallography and Photonics” of the Russian Academy of Sciences, Lenin Ave. 59, Moscow, Russia 119333; 5 Institute of Automation and Electrometry of the Siberian Branch of the Russian Academy of Sciences, Academician Koptyug Ave. 1, Novosibirsk, Russia 630090; 6 Novosibirsk State University, Pirogova Str. 2, Novosibirsk, Russia 630090; 7 Rzhanov Institute of Semiconductor Physics of the Siberian Branch of the Russian Academy of Sciences, Novosibirsk Branch “TDIAM”, Academician Lavrentyev Ave. 2/1, Novosibirsk, Russia 630090

Correction to: *Scientific Reports* https://doi.org/10.1038/s41598-021-02862-3, published online 06 December 2021

The original version of this Article contained an error in the Abstract.

“In this paper we describe the properties of the crystal of guanylurea hydrogen phosphate (NH_2_)_2_CNHCO(NH_2_)H_2_PO_3_ (GUHP) and propose its application in terahertz photonics and optoelectronics.”

now reads:

“In this paper we describe the properties of the crystal of guanylurea hydrogen phosphite (NH_2_)_2_CNHCO(NH_2_)H_2_PO_3_ (GUHP) and propose its application in terahertz photonics and optoelectronics.”

The original Article has been corrected.

