# Supplementary material for: A monoclinic semiorganic molecular crystal GUHP for terahertz photonics and optoelectronics
Source: Sci Rep. 2021 Dec 6;11:23433. doi: 10.1038/s41598-021-02862-3 (PMC8648782; doi:10.1038/s41598-021-02862-3)
Supplement: Supplementary file 1 — Supplementary Information. [file 41598_2021_2862_MOESM1_ESM.pdf]

Supplementary materials for "A monoclinic semiorganic molecular crystal GUHP for terahertz photonics and optoelectronics"

A Crystallographic characteristics, experimental data, and structure refinement parameters for GUHP

Chemical formula	$(NH_2)_2CNHCO(NH_2)H_2PO_3$ (GUHP)	
Space group, Z	Cc, 4	
Temperature (K)	293	80
a, b, c (Å)	6.6982(1), 6.8343(1), 16.3436(2)	6.6828(1), 6.7535(1), 16.2433(1)
β (deg)	96.5060(11)	96.5183(8)
V (Å^3)	743.351(18)	728.358(12)
D_x (g/cm^3)	1.645	1.679
Radiation; λ (Å)	MoKα; 0.71069	
μ (mm^{-1})	0.355	
Diffractometer	XtaLAB Synergy R, DW system, HyPix-Arc 150 Hybrid Pixel Array Detector	
Scan mode	ω	
T_{min}, T_{max}	0.906, 1.000	0.956, 1.000
θ_{max} (deg)	72.96	73.03
Ranges of indices h, k, l	$-17 \leq h \leq 17$; $18 \leq k \leq 15$; $21 \leq l \leq 30$	$-17 \leq h \leq 17$; $18 \leq k \leq 15$; $29 \leq l \leq 21$
Numbers of reflections: measured/ unique, R_{av}/ with I > 3σ(I)	20921/ 6093, 0.0094/ 4865	20570/ 5992, 0.0095/ 5879
R/wR	0.0224/0.0270	0.0136/0.0176
S	1.51	1.26
$\Delta\rho_{min}/\Delta\rho_{max}$ (e/Å^3)	-0.17/0.25	-0.14/0.19
Software	CrysAlis, Jana2006	

B THz TDS experimental set-ups

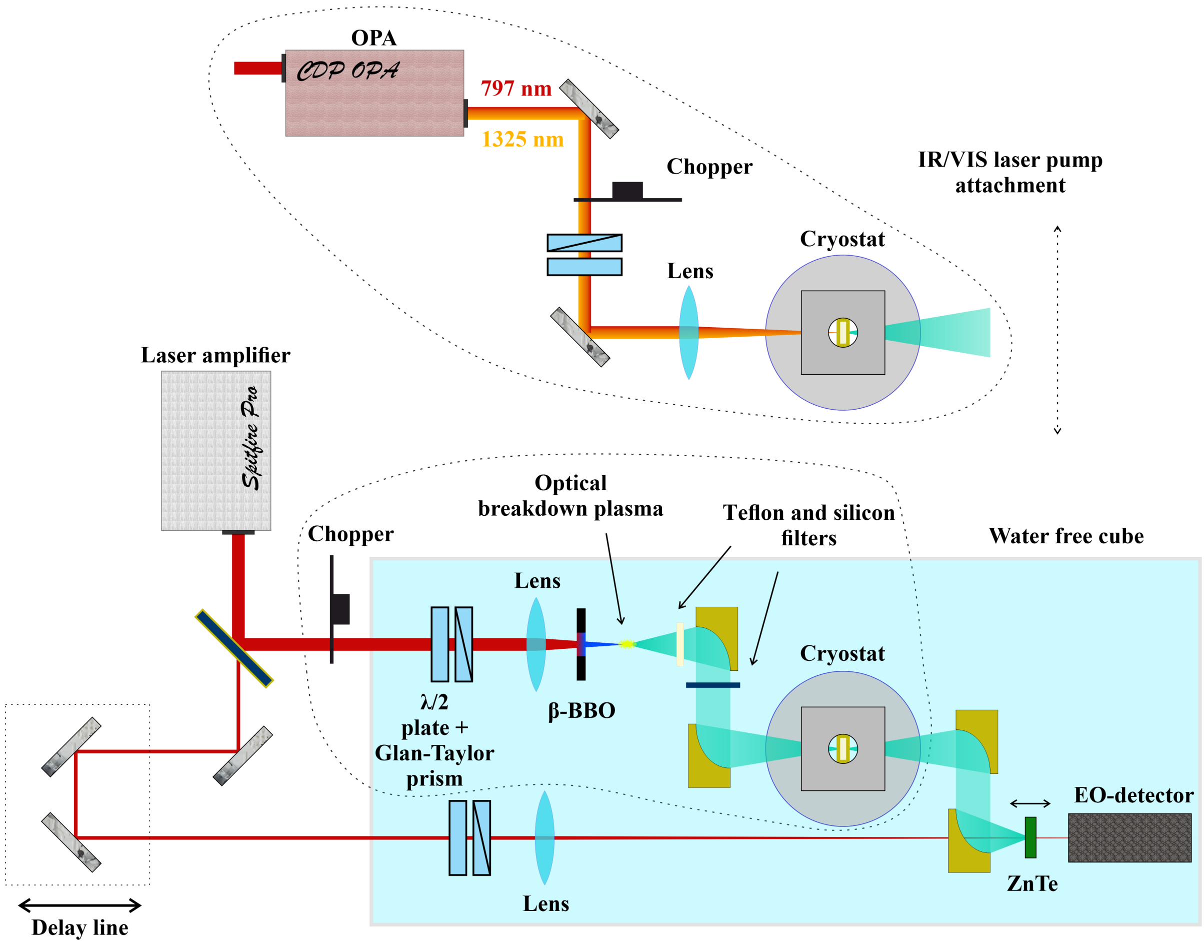

Experimental set-up of the THz time-domain spectrometer. An electro-optical system with a 1 mm thick ZnTe crystal was used as a detector of THz pulses. The effective spectral range recorded by this detector lies in the frequency range from 0.2 to 2.5 THz. A two-color optical breakdown plasma in air was used as a source of THz radiation. Cooling of the sample was carried out using a closed-cycle cryostat capable of cooling the sample to cryogenic temperatures. The pathway of the THz pulse was contained in a chamber filled with nitrogen gas, to reduce the attenuation of the THz signal by water vapor. For THz generation studies a Ti:sapphire regenerative amplifier or a two-stage optical parametric amplifier served to pump the crystal under study.

C Optimized fractional coordinates of unique atoms in crystallographic cell

T=293K			
Atom	x/a	y/b	z/c
P	7.783194995031E-02	2.979536046701E-01	-3.726918370565E-03
O	2.192633737333E-01	-8.460287645770E-04	2.314449298672E-01
O	5.566233399350E-02	3.640167304342E-01	8.475227249509E-02
O	-2.278226857176E-02	4.575972018217E-01	-6.924361388609E-02
O	-2.051383231520E-01	-2.393391109907E-01	-2.525308054349E-02
C	8.001834488150E-02	-5.515498687531E-02	1.794965414928E-01
C	-1.819599618401E-01	1.158257194800E-02	2.675709804859E-01
N	1.122214410730E-01	-1.293526101573E-01	1.061768811277E-01
N	-1.196087924325E-01	-4.353435648696E-02	1.943903528724E-01
N	-5.192025702842E-02	6.102875738129E-02	3.316355772571E-01
N	-3.769774380518E-01	1.251544571394E-02	2.731578939548E-01
H	-4.825602722832E-01	-2.145103406766E-02	2.252878471916E-01
H	6.996630539812E-02	-4.648599463623E-01	3.289981922460E-01
H	9.552877689309E-02	6.459978213627E-02	3.206989587201E-01
H	-1.038381706878E-01	1.212613102957E-01	3.829765803825E-01
H	2.596403901629E-01	-1.400735813262E-01	9.570878057505E-02
H	-6.826971323308E-04	-1.808176435311E-01	6.396711155089E-02
H	-2.342935625782E-01	-8.723515219589E-02	1.489745085504E-01
H	3.972149044754E-01	7.270295035043E-02	-4.775269239534E-02
H	-3.627607680311E-02	1.257764878655E-01	-2.295110357407E-02
T=80K			
Atom	x/a	y/b	z/c
P	7.971802851065E-02	3.075947326493E-01	-3.687772464463E-03
O	2.183317638878E-01	-1.156227578811E-03	2.318423178037E-01
O	5.693567614008E-02	3.705242145627E-01	8.450422770062E-02
O	-1.788265312925E-02	4.685771521231E-01	-6.817424118978E-02
O	-2.039345497479E-01	-2.311822881604E-01	-2.501582796014E-02
C	7.921298911157E-02	-5.213351516125E-02	1.797004272560E-01
C	-1.824388844993E-01	9.842230594181E-03	2.677713766923E-01
N	1.111933880774E-01	-1.216050927043E-01	1.061257744363E-01
N	-1.201894391748E-01	-4.146108072869E-02	1.946118069061E-01
N	-5.256484477930E-02	5.569922454197E-02	3.318817695645E-01
N	-3.772068845019E-01	1.085145378083E-02	2.733414672818E-01
H	-4.828170765081E-01	-2.048329828412E-02	2.255390630505E-01
H	7.033380838953E-02	-4.703303291787E-01	3.291581437311E-01
H	9.469662056301E-02	5.892747536893E-02	3.210762372703E-01
H	-1.040132137819E-01	1.136424464643E-01	3.833297363902E-01
H	2.582682224979E-01	-1.318961138834E-01	9.563118581939E-02
H	-1.487420410417E-03	-1.707013309091E-01	6.368956165037E-02
H	-2.346719415502E-01	-8.258706742614E-02	1.490244776776E-01
H	4.011127374634E-01	8.117203965744E-02	-4.689727116071E-02
H	-3.629632655818E-02	1.390579251086E-01	-2.403846045572E-02
